# An alternative approach to implementing patient-reported outcome measures

**DOI:** 10.1186/s40814-018-0289-1

**Published:** 2018-07-04

**Authors:** Elizabeth Gibbons, Ray Fitzpatrick

**Affiliations:** Health Services Research Unit Nuffield Department of Population Health, University of Oxford, Richard Doll Building, Old Road Campus, Oxford, OX3 7LF UK

**Keywords:** PROMs, Musculoskeletal, Implementation, Impact, Service improvement, NHS, Physiotherapy

## Abstract

**Background:**

Obtaining patients’ views of their health and outcomes of interventions utilising patient-reported outcome measures (PROMs) is a well-established method, but there is still uncertainty about the impact of PROMs on services and patient care. Studies are now needed of alternative ways of implementing PROMs. This paper describes a case study of the introduction of a new PROM to assess musculoskeletal (MSK) problems, known as the Musculoskeletal Health Questionnaire (MSK-HQ).

**Methods:**

Following an invitation from the Arthritis Research UK (ARUK), 11 groups and organisations agreed to become ‘partners’ in piloting the MSK-HQ. Twenty-nine interviews and a focus group were carried out with key informants from the partners. Interviews were supplemented with some documentary evidence of partners’ meetings. Data were coded and analysed with NVivo software V.10. Analysis was carried out via a framework method.

**Results:**

Participants reported positive evidence that the MSK-HQ is feasible and practical for use in patient care with content that helped health professionals identify and address patients’ main presenting problems. Although mediated and reported through health professionals’ judgments, the questionnaire was also seen as very relevant and acceptable to a wide spectrum of patients.

There was also broad support for the view that whilst the MSK-HQ is relevant to individual patient care, it could also, when aggregated, reflect the experiences of patients as a group and be used as evidence for third parties concerned with the provision and commissioning of services.

The main difficulties revealed by the case study were in the form of logistics and sustainability. It was recognised that electronic systems would be more effective for administration and data processing but they were not feasible to develop and implement within reasonable timelines and available budgets. A sustainable approach to using the PROM required significant long-term commitment of budget, a coherent system, and active support from diverse organisations.

**Conclusions:**

The current study supports the view that a bottom-up approach is a promising method to generate PROM-related insights that are relevant to patients and health professionals. The partnership approach to developing and using PROMs may have wider relevance and potential as a model of implementation.

**Electronic supplementary material:**

The online version of this article (10.1186/s40814-018-0289-1) contains supplementary material, which is available to authorized users.

## Background

Patient-reported outcome measures (PROMs) are well-established, validated methods of obtaining patients’ views of their health and outcomes of interventions. They have great potential to improve the responsiveness of health services. To date, there is mixed evidence of their actual impact. There is some promising evidence, from experimental trials, that, at the micro-level of individual patient care, PROMs can improve communication between patients and health professionals and facilitate better outcomes but not all evidence points in that direction [1]. It is not clear whether PROMs used in individual patient care are compatible with their aggregated use to assess system and service-level performance in order to improve the quality of services [2, 3].

One of the most ambitious programmes to introduce PROMs was carried out in the National Health Service (NHS) in the form of a centrally coordinated and nationally mandated initiative to monitor patients undergoing selected elective surgical procedures (hip and knee replacement, hernia and varicose vein surgery) with a view to providing aggregate-level evidence of the performance of services [3]. It has produced a wealth of information about needs, outcomes and surgical performance in relation to the four procedures selected. However, there is growing uncertainty about the overall impact of the PROM programme on the quality of services and about optimal methods of implementing PROM programmes [4]. Evidence from this programme and related initiatives suggests that if health professionals are insufficiently engaged at the outset, and are unclear about the meaning and value of information from PROMs, this may reduce the potential impact of this new form of evidence about outcomes [4, 5].

The basic science of PROMs as a method of measurement is well developed. The main uncertainties now are about whether and how PROMs can positively impact on services [6]. To address this uncertainty, studies are now needed of alternative ways of implementing PROMs [7]. This paper describes a case study of the introduction of a new PROM to assess musculoskeletal (MSK) problems, known as the Musculoskeletal Health Questionnaire (MSK-HQ) [8]. The MSK-HQ was developed with extensive patient and health professional involvement. In addition to containing questions commonly found in such measures, for example about pain and function, the 15-item questionnaire asks respondents about how well they feel they understand their condition and how confident they are in dealing with symptoms. Its development was sponsored by a UK-based charity, Arthritis Research UK (ARUK), that hoped that the new instrument might be applicable across the widest possible spectrum of musculoskeletal problems, to support the charity’s mission to improve the quality of life of individuals with MSK disorders.

The aim of the study was to identify any lessons from a case study of the piloting of MSK-HQ about factors promoting or inhibiting successful adoption of a PROM in health services.

## Methods

An invitation was issued by the ARUK for clinical groups or healthcare organisations to pilot the MSK-HQ. The offer in the invitation was that they would be provided basic advice and information about the PROM if they volunteered to use it but it would be expected that they share their experiences and insights (positive and negative) from using the PROM with each other by being interviewed twice (at baseline and follow-up at 1 year) in the course of a year-long project, facilitated by the authors of this study. No resources from the charity were provided to support the pilots.

Eleven groups and organisations agreed to become ‘partners’ in this arrangement (briefly summarised in Table 1). For this study, 29 interviews were conducted with key informants from the partners, mostly via telephone and recorded with participants’ permission. In addition, a focus group was carried out with participants from seven of the partners. Interviews were supplemented with some documentary evidence of partners’ meetings (Additional file 1: Table S1).

**Table 1:** Description of partner organisation

Service	Participants	Drivers	Methods of administration
Hospital sports and exercise medicine	SEM, consultant	No PROM data currently collected. Essential for revalidation	Paper-based completion in clinic
Community physiotherapy department (GP referrals/hospital discharge rehabilitation)	Physiotherapists (*n* = 7) and service lead	To provide evidence to CCG for re-commissioning of service, competitive benchmarking and audit	Paper-based completion in clinic
Hospital physiotherapy department and ankylosing spondylitis clinic	Physiotherapist and AS lead	Individual patient care but feedback to GPs and CCG	Paper-based completion in clinic
Chronic pain clinic: pain management course	Service lead and clinical psychologist	Baseline and completion following pain management course	Paper-based completion in clinic
Osteopathy (non-NHS)	Vice principal of research and senior osteopath	Clinical monitoring and provide aggregate data for benchmarking. Present data stakeholders—the Board and institution, faculty members, students, patients and General Medical Council	Paper-based administration and electronic scan using optical recognition software. Link with an ID to the patient’s clinical electronic record and generate an automated email at 6 and 12 weeks
MSK services	Programme lead	Aggregate data for benchmarking and service improvement	Remote electronic data capture baseline and 3 months
Integrated MSK service	GP commissioner, patient partner lead and quality manager	CCG and for benchmarking with other services providers and for specific patient pathways of care	External company employed to capture and process data. Postal paper-based and electronic via text and/or email
Spine pathway	GP with special interest in MSK; senior physiotherapist, GP	Aggregate data to compare between clinicians and interventions and CCG requirement	Postal questionnaire (by email or text) 10 days before appointment and entry in to service then following discharge (3 months)
Intermediate diagnostic MSK service	Specialist service manager, GP commissioner	Aggregate data to feedback to managers and commissioners	Paper-based completion in clinic
Hospital physiotherapy/occupational health	Physiotherapy and physiotherapy-led orthopaedic triage clinics. Primary and secondary care patients	Patient-level data to inform clinical care and aggregate data to feedback to team members	Paper-based completion in clinic
Musculoskeletal pain and podiatry	Clinical physiotherapy specialists and service improvement teams	Implement as part of quality improvement programme. Patient-level data to inform clinical care and aggregate to improve services	Exploring integration with clinical systems

Data were coded and analysed with NVivo software V.10. Analysis was carried out via a framework method of analysis suitable for applied health research, permitting the use of both inductive insights from the data and deductive themes previously identified from research on PROMs [9].

## Results

### Positive views about the new PROM

#### Relevance to practice

Generally, respondents were positive about the relevance of the content of the MSK-HQ to their clinical work; the instrument spoke to what they saw as their professional role in individual patient care.

So it was those sort of extra domains that we felt we could quite capture some of the stuff that we achieve. So just looking through the questions it looks like a good measure really to capture what we do on a day-to-day basis. (Community physiotherapist, clinical lead)

Capturing psychosocial aspects of patients’ quality of life was considered an attractive feature of the measure. Where problems were identified, this was thought to be useful to address at an individual level with the patient or potentially to provide evidence for referral to other services.

Because there are a couple of things around mental health as well, especially for physiotherapists I think it’s sometimes a difficult subject to broach and if you have – oh you scored relatively low on this question, can you tell me a little bit more about why that is? (Spine pathway, Consultant physiotherapist)

Furthermore, the inclusion of items capturing patients’ understanding of their condition and confidence to self-manage was thought novel and valuable. Patients’ understanding of their condition was considered to be crucial to the overall outcome of treatment.

We were also quite attracted to; there were questions on there about a patient’s understanding of their condition and if they are self-managed. So if we’ve got something that we perhaps are not going to change, like an arthritic knee, or something like that, you know at least we thought well actually we can affect their emotional well-being. We can improve their understanding and their self-efficacy. (Community Physiotherapist, clinical lead)

Specific items were considered important for identifying areas of unmet need and potentially expanding the scope of their practice or referral to other services. For example, the questionnaire item on the level of physical activity item was considered to be useful as a stand-alone indicator to demonstrate impact of treatment on activity but also meet the requirements of the NICE Quality Standard.

Yes so we could keep that separate as well, because the other thing I was thinking was, so we can say this is the PROM and then with this one there is a NICE quality standard, so we could just use that as a separate piece of data. And we could use them in two different stats. We could say this is our change and this is how we can increase their activity levels by coming to physio and use that piece of data in a different way. (Community physiotherapy team)

Other examples were given relating to identifying the impact of the patient’s condition on sleep and anxiety. This could for example lead to further discussion and targeting of treatment.

With some of the questions and if you analyse the form with the patient, once the patient has filled it out, then you can say, oh yes we can see here that you are having trouble with your sleeping and you are getting quite anxious as well. You can then pick out little things that can guide you with your treatment. Target them. And then the patient can then say, oh yes I can see the link between this. Maybe if we help to sleep, it might help you become less anxious and help with your pain and things. So we are identifying that as kind of a learning, so I have contacted a sleep expert and had discussions and he is going to do a 2 – 21/2 hour talk to all my staff now about how we can address some of the sleep issues. (Spine pathway, Consultant physiotherapist)

There was some suggestion that PROM data might be useful to indicate that some specific patient populations do not benefit from referral to services. Although challenging to professional views, this could be fed back to GPs and signpost the need for different interventions or services.

Yes, but in regards to the MSK-HQ, if a doctor refers a patient and we treat that patient and we feel that they are not improving and the questionnaire suggests the same. They’ve not improved but they’ve done well from their questionnaire, at least we’ve got evidence to go back to the GP and say we’ve been seeing your patient and actually it’s not making any difference. You wonder whether they would benefit from a different service. (Community physiotherapy team)

It was thought that the questions relating to patients’ understanding of their condition and their confidence in managing symptoms were relevant to clinicians’ responsibilities to educate patients and could potentially highlight areas of further training needed for clinicians. Furthermore, PROM data were considered useful for benchmarking and comparing outcomes for care provided by individual clinicians and different services.

The spine pathway lead is engaged in when we break it down by clinician, potentially there can be some feedback that can go out and that’s something that’s been discussed and is going to be done. So actually I think we have made it clear that we are going to be looking at it being broken down by clinician and I think we have to deal with that sensitively. So we are kind of trying to build in that it’s not about judging people, it’s about working. (Spine pathway, Consultant physiotherapist)

So you know are we treating shoulder patients better than our knee patients. Or is this clinician finding shoulders easier to treat, or getting a better outcome with shoulders than that clinician. (Community physiotherapist)

#### Patient acceptability

The partners reported high level of patient engagement with the measure, no reports of patient refusal to complete and informal feedback from patients who suggested it was relevant to their condition, easy to complete and preferred to other measures they had completed in the past. Patients in one site were cited as preferring to use the MSK-HQ compared to other more lengthy measures that they were also invited to complete.

So pretty much everybody has said it’s easier -one person said it’s better because it’s not intrusive it’s not as personal as the other ones. It’s getting to the right areas, but it’s not going so deep [informal feedback from patients to staff. (Psychologist, chronic pain)

One partner organisation conducted a survey of *n* = 68 patients obtaining their views of the content and acceptability of the MSK-HQ and relevance for clinical care.

Most patients found all the questions applicable and easy to complete. Paper-based completion in the clinic was most popular but most would prefer completion by email at follow-up. (Consultant, Sports and Exercise Medicine)

#### Relevance to funders

Respondents were generally very positive about the results from the MSK-HQ being available to meet demands from commissioners to demonstrate services were benefiting patients as well as identifying areas for service improvement.

Driven by the need to argue my case for funding or whatever, then I can say that we’ve got evidence to show that we do things well and we make a difference. (Community physiotherapy, service lead)

Yes, so I think as I was kind of saying, now with the NHS, NHS physio and the commissioners and change in services and things and also patient’s expectations, it’s really important that we can show how well we are doing, or for areas of improvement of as well. So we can ensure that we are working at our very best. (Community physiotherapist)

The PROM provided staff with data that could be relatively quickly provided for commissioners. Often, such data is requested from commissioners at short notice.

We are very aware that people can be given quite short notice to produce something. So that’s why the group started to think of pre-planning because some of the managers are being put in a position where they say – right let’s do this now, can you please provide a data breakdown? Obviously it takes a long time to collect that. It is sort of pre-empting of, well we have got something. (Community physiotherapist, clinical lead)

Again, it was thought that specific questionnaire items in the measure were of particular importance to commissioners of services particularly ‘shared decision making’ and ‘health literacy’. These are considered important indicators of quality.

There are requirements from the CCG to demonstrate that patients are being involved in decision making; there has been an improvement on their understanding of their condition. (Hospital physiotherapist)

### Challenges

#### Interpretability

Although there was widespread enthusiasm for the measure in terms of its content, there were other concerns, particularly about the interpretation of a new measure. For example, the MSK-HQ asked respondents about a 2-week interval for someone with chronic pain which was considered too short. It was not clear that it was the relevant time frame for patients with chronic pain*.* More generally, there was concern as to whether the new PROM would be sufficiently sensitive to change.

That was one of my worries was it going to be sensitive enough for chronic conditions and the other thing was how do I compare my service with somebody else. (Spine pathway, Consultant physiotherapist)

In several contexts, staff and commissioners required other PROMs to be used and would need persuading of the merits of changing to MSK-HQ.

I think I’ve always believed that myself, but yes I guess staff need to be on board with that as well and in all honesty I think the more there is published research on MSK-HQ comes out the better. You know to see how well the MSK-HQ is performing against the disease specific. (Hospital physiotherapy clinical lead)

In many ways, concerns from partners were focused on the general issue of how to interpret the scores, especially change scores.

The responsiveness side of it to me is probably the most important thing at this stage. Because, if we can prove it’s as responsive as some of the other disease specific measures, then we can kind of start radically changing what we are doing. We can stop collecting certain measures, and we can just collect the MSKHQ and suddenly you have eased the burden on staff and things are changing. (Hospital senior physiotherapist)

One specific challenge highlighted was how to interpret item-level changes which were not apparent in the summed score. This supported the view that individual items were in some cases more relevant to clinical care and to commissioners.

Someone may improve on one section but not on another and having a score out of ……however many it is, doesn’t really show which bits improved. (Hospital Physiotherapist, clinical lead)

Partners recognised the potential value and relevance to commissioners of the new MSK-HQ but were concerned as to how well they would be able to interpret data from the new instrument:

So I think, certainly from a commissioner’s perspective I am very conscious that we have to be cautious not to use any single source of data, because of kind of gathering the data comprehensively from within the pathway or even geographically. You know, I think one has to fear, as one of the tools that we use to triangulate the quality of our service. I would be very dubious about judging the quality of a service based purely on one PROM. (Commissioner, Spine pathway)

#### Feasibility and sustainability

There were concerns about how the new PROM could be integrated into current practice. One partner site planned for administrative staff to administer the MSK-HQ in the clinic prior to the appointment. For others, there was concern that it would not be easy to integrate administration of the questionnaire with the routines of the consultation. It would be a change in the way that the health professional worked.

I think for me that would require a big shift to use the questionnaire as a thing and then to score it further. Because I am used to using a more narrative approach, the patient’s story and delve in where it’s appropriate. I know that some of them are intending to use them that way, but that would be quite hard for me I think. (Community physiotherapist, clinical lead)

Even more challenging was how to determine the timing and mechanism of follow-up administration of the PROM. Alternatives were either to aim for standardised timing, for example as close to 3 months after baseline as possible. Conversely, the timing could be determined by attendance at clinics.

What we’ve decided in terms of baseline and follow up is we are going to do our baseline invites and we will do a follow up a three months, regardless of where people are in the system, because there are discussions around should we vary it, should we have our baseline and then on discharge. Discharge could be quite a long time for one person and very swift for another, so we just went with a standard and then three months. (MSK programme Lead)

Longer term follow-up was also considered to be ideal, but partners foresaw the challenges of collection in terms of losses to follow-up and resource implications. Patients experience a wide diversity of pathways after any given treatment, and tracking such diversity was seen as challenging given current medical records and information technology.

Not many are still with us at three months. In terms of funding and sending these questionnaires out at six months and twelve months, it would be great to get that data, but that would involve quite a shift from where we are. Even implementing it as an outcome measure is quite a lot from where we are now. (Pain and podiatry physiotherapist)

There were discussions within partner organisations about the potential to integrate the PROM into electronic clinical systems. This was particularly challenging for some sites as new clinical systems were being implemented across the patch notwithstanding the technical aspects of incorporating additional features in the software.

Yes. I think all these electronic templates, they can be quite complicated, you only have to make a small error on the template and it becomes more difficult to pull the data. (Hospital senior physiotherapist)

But that’s the bit we are struggling with. But it’s finding a way to input it in such a way that we can extract the data easily, with minimal input from somebody having to manually input the data afterwards. So it’s trying to find a way that patients’ answers go straight into a system, but then when they do it a second or third time, so that the data will be extracted, so we are trying to’ get that bit right now. (Pain and podiatry, physiotherapist)

Nonetheless, electronic capture of data was seen as the ideal method, especially if incorporated into clinical systems. Linking across settings was not without logistical challenges, particularly across primary care and community and secondary care, as electronic records and systems were either incompatible or in many cases, especially in secondary care, paper records were the only method.

Yes, that’s the conversations we are having and are waiting to see the outcome of this piece of work, because our MSK leads are very positive about it, if we can find a way to embed it electronically, it will make life easy for them then that’s a bonus. (MSK programme Lead)

So it’s slightly different, you know, the whole trying to get something new set up there is very difficult to try and get something set up across the community where it needs to be electronically smarter. That’s the biggest barrier actually, so it’s getting over that barrier that’s going to be the key. (Hospital senior physiotherapist)

Capturing patients in low deprivation areas without access to Internet for electronic capture was also considered problematic. One partner lead was implementing the measure electronically across several health boards but experienced complex discussions and complications regarding data governance and privacy issues especially as external providers were contracted to collect data.

There has been a lot of background work required in terms of privacy impact assessment and governance arrangements within each of the health board, each of those is at a varying state of governance about use of email for clinical contact with patients. (MSK programme Lead)

Generally, data processing in relation to the PROM was seen as difficult with little or no additional or dedicated staff being available to support the innovation.

A more general concern focused on the longer term sustainability of collection of the new PROM, particularly in terms of funding for infrastructure to collect and process the data.

We don’t know as yet, we do have a system that we might be able to apply to, or to see whether actually it would be financed through the existing budget. We don’t know yet is that answer to that. (Pain and podiatry, physiotherapist)

Staff engagement was viewed as crucial to continued data collection, and different methods were planned to support and enhance engagement. One partner site planned to conduct interviews with administrative staff to assess the impact on their time and suggestions for further implementation. Staff training was planned in some partner sites.

I have kind of toyed with it for other reasons, I have got some videos and some online stuff around consent and communication and consultations. I toyed a little bit with, you know, could we develop a brief little online something to help people get their heads around it and engage with it. (Osteopathy)

## Discussion

This case study provides positive evidence that a newly developed and introduced PROM can address needs in patient care. This approach by case study complements the very important body of evidence from studies in the form of experiments to examine the impact of PROMs on outcomes but where issues of external validity may arise. The MSK-HQ was widely seen as being both feasible and practical for regular use in patient care with content that helped health professionals identify and address patients’ main presenting problems. Although mediated and reported through health professionals’ judgments, the questionnaire was also seen as very relevant and acceptable to a wide spectrum of patients.

There was also broad support that the same instrument, whilst relevant to individual patient care, could also, when aggregated to reflect the experiences of patients as a group, be used as evidence for third parties concerned with the provision and commissioning of services.

The main difficulties revealed by the case study were in the form of logistics and sustainability. Introducing even a simple questionnaire into a clinical service proved a management challenge. Even with extensive enthusiasm and support from health professionals, it proved difficult to create a reliable system to obtain MSK-HQ results from all relevant patients and then make them available to support clinical and other decisions. The various clinical groups in the case study with few exceptions resorted to traditional methods of data collection via low-technology paper-based administration. It was recognised that electronic systems would be more effective but they were not feasible to develop and implement within reasonable timelines and available budgets. The related concern that was widely identified was sustainability. A sustainable approach to using the PROM required significant long-term commitment of budget, a coherent system and active support from diverse organisations. This commitment could not be guaranteed given the bottom-up way in which the MSK-HQ had been introduced and piloted into a variety of settings.

The most distinctive feature of the launch of the MSK-HQ was the sense of engagement and partnership between health professionals, patients and the advocacy from a health charity in recognising the need for a PROM to work across musculoskeletal health. These three groups worked together collaboratively to help develop the instrument and then to test it out in the real world of clinics and services. The recognition of the potential value of the PROM by clinicians in particular found in this study is in contrast with evidence that health professionals often struggle to see the validity, relevance or value of PROMs [10]. Similarly, the observation that health professionals were comfortable with the use of data from PROMs by third parties is in contrast with the view that aggregated use of PROMs to inform decisions about quality and performance is problematic for health professionals [2, 10]. As commonly found, the main challenge observed in this case study stems from the many limitations of current medical information systems [2, 6, 11]. This is likely to be a healthcare system-level problem; evidence is emerging of other systems more effectively integrating PROMs into routine IT supporting services [12].

This partnership model is in clear contrast to initiatives such as the NHS national PROM programme which was centrally driven using substantial public resources to require implementation of PROMs to monitor patients undergoing certain elective surgical procedures. It has been argued that this top-down approach, whilst providing very substantial resources for the delivery and collection of responses from PROMs, failed to engage with clinicians and professionals about appropriate methods or how to disseminate and use results [4]. The current study supports the view of Kyte and colleagues that a bottom-up approach is more likely to generate PROM-related insights that are relevant to patients and health professionals [4]. At the very least, the bottom-up approach may provide momentum and support for the introduction of a PROM that subsequently requires broader coordination ‘from above’. Others have also recently argued for the potential importance of a bottom-up approach in influencing the adoption of PROMs [13].

There are limitations to the current study. The only feasible method to study the introduction and voluntary adoption of a PROM across a spectrum of very varied organisations was an observational case study, largely relying on interviews, with no scope for experimental design. The very heterogeneity of organisations studied may also mean that unobserved factors might have been important influences. Also, the study design resembles action research in that the researchers had links to the MSK-HQ and to the ARUK and so may have had biases in their interpretation of evidence from the case study.

The model whereby the different parties concerned with a long-term condition come together to generate mutually relevant evidence via a PROM may have broader significance. Many other long-term conditions raise issues about how the individual with the condition and his or her health professionals make decisions about how best to live well with the condition. PROMs are a key resource to support such decisions [14].

## Conclusion

The partnership and bottom-up approach to developing and using PROMs may have wider relevance and potential as a model. The current study supports the view that a bottom-up approach is a promising method to generate PROMs-related insights that are relevant to patients and health professionals.

## Additional file

Additional file 1: **Table S1.** Description of partner organisation. (DOC 35 kb)

## Abbreviations

ARUK: Arthritis Research UK
MSK: Musculoskeletal
MSK-HQ: Musculoskeletal-Health Questionnaire
NHS: National Health Service
PROMs: Patient-reported outcome measures
