# Supplementary material for: An alternative approach to implementing patient-reported outcome measures
Source: Pilot Feasibility Stud. 2018 Jul 4;4:96. doi: 10.1186/s40814-018-0289-1 (PMC6031120; doi:10.1186/s40814-018-0289-1)
Supplement: Supplementary file 1 — Table S1. Description of partner organisation. (DOC 35 kb) [file 40814_2018_289_MOESM1_ESM.doc]

**Table S1: Description of partner organisation**

| **Service** | **Participants** | **Drivers** | **Methods of administration** |
| --- | --- | --- | --- |
| Hospital Sports and Exercise Medicine | SEM, Consultant | No PROMs data currently collected. Essential for revalidation | Paper-based completion in clinic |
| Community physiotherapy department (GP referrals/ hospital discharge rehabilitation) | Physiotherapists (n=7) and Service lead | To provide evidence to CCG for re-commissioning of service, competitive benchmarking and audit | Paper-based completion in clinic |
| Hospital physiotherapy department and Ankylosing Spondylitis clinic | Physiotherapist and AS lead | Individual patient care but feedback to GPs and CCG | Paper-based completion in clinic |
| Chronic pain clinic: Pain management course | Service lead and clinical psychologist | Baseline and completion following pain management course. | Paper-based completion in clinic |
| Osteopathy (non-NHS) | Vice Principal of Research and Senior osteopath | Clinical monitoring and provide aggregate data for benchmarking. Present data stakeholders- the Board and institution, faculty members, students, patients and General Medical Council. | Paper based administration and electronic scan using optical recognition software. Link with an ID to the patient’s clinical electronic record and generate an automated email at 6 weeks and 12 weeks. |
| MSK services | Programme lead | Aggregate data for benchmarking and service improvement | Remote electronic data capture baseline and 3 months |
| Integrated MSK service | GP commissioner, Patient partner lead and Quality manager | CCG and for benchmarking with other services providers and for specific patient pathways of care | External company employed to capture and process data. Postal paper-based and electronic via text and/or email. |
| Spine pathway | GP with special interest in MSK; senior physiotherapist, GP | Aggregate data to compare between clinicians and interventions and CCG requirement | Postal questionnaire (by email or text) 10 days before appointment and entry in to service then following discharge (3 months) |
| Intermediate diagnostic MSK service | Specialist service manager, GP commissioner | Aggregate data to feedback to managers and commissioners | Paper-based completion in clinic |
| Hospital physiotherapy/ Occupational Health | Physiotherapy and Physiotherapy led Orthopaedic Triage Clinics. Primary and secondary care patients | Patient level data to inform clinical care and aggregate data to feedback to team members | Paper-based completion in clinic |
| Musculoskeletal pain and podiatry | Clinical physiotherapy specialists and service improvement teams | Implement as part of quality improvement programme. Patient level data to inform clinical care and aggregate to improve services | Exploring integration with clinical systems |
